# Clinical Challenge: Patient With Severe Obesity BMI 46 kg/m^2^

**DOI:** 10.3389/fendo.2019.00635

**Published:** 2019-10-02

**Authors:** Gitanjali Srivastava, Caroline M. Apovian

**Affiliations:** Section of Endocrinology, Diabetes, Nutrition and Weight Management, Department of Medicine, Boston Medical Center, Boston University School of Medicine, Boston, MA, United States

**Keywords:** anti-obesity medications, weight loss drugs, combination therapy, bariatric surgery, lifestyle intervention

## Abstract

Obesity causes and exacerbates many disease processes and affects every organ system. Thus it is not surprising that clinical providers are often overwhelmed with the multitude of symptomatology upon initial presentation in patients with obesity. However, despite a “complicated medical history,” a systematic, organized approach in obesity medicine utilizes a personalized-tailored treatment strategy coupled with understanding of the disease state, presence of comorbidities, contraindications, side effects, and patient preferences. Here, we present the case of a young patient with Class 3b severe obesity, several obesity-related complications, and extensive psychological history. Through synergistic and additive treatments (behavioral/nutritional therapy combined with anti-obesity pharmacotherapy and concurrent enrollment in our bariatric surgery program), the patient was able to achieve significant −30.5% total body weight loss with improvement of metabolic parameters. Though these results are not typical of all patients, we must emphasize the need to encompass all available anti-obesity therapies (lifestyle, pharmacotherapy, medical devices, bariatric surgery in monotherapy or combination) in cases of refractory or severe obesity, as we do similarly for other disease modalities such as refractory hypertension or poorly controlled Type 2 diabetes that requires robust escalation in therapy.

## Clinical Challenge

A 31 year old patient with a past medical history of Class 3 obesity BMI 46 kg/m^2^, Type 2 diabetes mellitus (A1c <5.7%, well controlled on metformin), polycystic ovarian syndrome, non-alcoholic steatosis of the liver, pulmonary and neurosarcoidosis on infliximab and methotrexate, and chronic worsening pain presents for weight management evaluation. She had a history of opioid use disorder due to the chronic pain, though in remission. She had been on several weight-promoting pain medications for symptom control, including gabapentin, duloxetine and nortriptyline. Contributing factors over the years to her weight gain also included her diagnosis of Bipolar Disorder with antipsychotic medication-induced weight gain (previously trialed aripiprazole, responded to lurasidone with decreasing efficiency, and now finally stable on paliperidone though weight gain promoting). Her highest adult weight was her current weight of 295 pounds with a lowest adult weight of 140 lbs. that pre-dated her Bipolar and sarcoidosis diagnoses several years ago. She had stable eating patterns, and often chose healthy meals such as hummus, vegetables, Greek salads, and lean meats, though had a weakness for sweet cravings. She engaged in structured gym exercise for 30 minutes three times per week despite the chronic pain. Recent stressors included her close aunt who had been diagnosed with cancer. She also suffered from insomnia and had been evaluated closely with sleep therapists and sleep hygiene specialists. Her polysomnogram was negative for sleep apnea.

## What Would You Do Next?

- Offer more aggressive intensive lifestyle therapy intervention
- Trial of anti-obesity medication if option A above becomes ineffective
- Metabolic and bariatric surgery only as anti-obesity medication would be contraindicated given her history of opioid use
- Trial of anti-obesity medication for 3 months with concurrent referral to bariatric surgery

## Discussion

The patient depicted in the case has chronic, debilitating severe obesity classification with several inflammatory obesity-related comorbidities and other contributing etiology to her weight gain.

In regards to lifestyle intervention, the patient was started on a healthy low fat high fiber diet with increased consumption of vegetables, while minimizing intake of processed foods, added sugar, *trans* fats, and refined flours (1). Nutrient-dense whole foods prepared at home were encouraged. Acceptable macronutrient distribution range is 45–65% carbohydrates, 20–35% total fat of which <10% should be polyunsaturated fats, and 10–35% protein and amino acids^1^. However, obesity-related comorbidities such as type 2 diabetes mellitus, polycystic ovarian syndrome, and non-alcoholic steatosis of the liver suggesting features of insulin resistance need to be taken into consideration when implementing dietary modifications specific to this case. The patient's daily carbohydrate intake should be reduced to 40–50% to combat insulin resistance. Several studies have shown improvement in metabolic parameters and more rapid weight loss when a low carbohydrate diet was implemented initially in the first 3–6 months (2, 3). At presentation, the patient's calculated daily protein intake was <20% of total daily intake and increasing her protein intake to 30% reduced her sweet cravings and increased satiety. In addition, she would benefit from at least 150 min per week of structured moderately intensive exercise as tolerated as recommended by The American College of Sports Medicine (4). Of note, the patient is also under significant stressors. Stress has been very strongly linked to hyperphagia, binging, and obesity (5, 6). Stress management would also provide long-term strategies for emotional/stress eating should they arise. Her sleep has been adequately addressed by a specialist multidisciplinary team. Further, the patient was already under intense behavioral therapy given her underlying psychiatric illness. Early behavioral therapy intervention should be strongly considered in patients with adverse psychological factors, eating disorders and underlying psychiatric conditions that would otherwise impede their overall progress toward health goals. However, it may be difficult to promote more aggressive lifestyle intervention alone, especially in a patient with an advanced obesity disease staging who is already making strides to eat healthy and undergoing behavioral therapy.

Furthermore, the patient also meets criteria for initiation of anti-obesity pharmacotherapy (AOM): BMI >27 kg/m^2^ plus the presence of one obesity-related comorbidity and/or BMI >30 kg/m^2^ in conjunction with lifestyle intervention (7, 8). Though the patient has a history of opioid use disorder, it is in remission and there is no active contraindication to AOM. The patient also does not have underlying heart disease, end-stage-renal disease, or acute angle glaucoma that would negate use of several AOM such as phentermine/topiramate, lorcaserin, and naltrexone/bupropion. Liraglutide 3.0 mg would be a first option given its double benefits in patients with severe obesity and diabetes (7) and other obesity-related comorbidities such as fatty liver (9) and polycystic ovarian disease (10). The medication is also generally well-tolerated and safe. Because anti-obesity medications can exert central effects in a patient with Bipolar Disorder, close monitoring and communication with the patient's psychiatrist would be critical. Because her BMI is already very elevated, clinically, both lifestyle changes and pharmacological treatment would be implemented together, rather than separately. Moreover, based on her current body mass index alone of 40 kg/m^2^, the patient meets National Institutes of Health consensus criteria for metabolic and bariatric surgery (11): BMI 35 kg/m^2^ in the presence of at least one obesity-related comorbidity or BMI 40 kg/m^2^. Therefore, it would be prudent to discuss bariatric surgery in this patient given her disease severity.

The correct answer is D. The patient was actually started on AOM with concurrent referral to the institution's bariatric surgery program. Since the patient's insurance did not provide coverage for liraglutide 3.0 mg, she was alternatively prescribed a combination anti-obesity medication therapy (phentermine/topiramate) after discussion with her psychiatrist and other specialists. AOM were instrumental in improving the patient's overall hunger drive, cravings, and satiety. Despite being the best option for her at presentation, the patient was unwilling to undergo the bariatric procedure. Oftentimes, this may be the case in many patients until they consent to surgical intervention or have weight regain on non-surgical therapy. Future guidelines may need to be more definitive about earlier referral to bariatric surgery.

**Figure 1:**
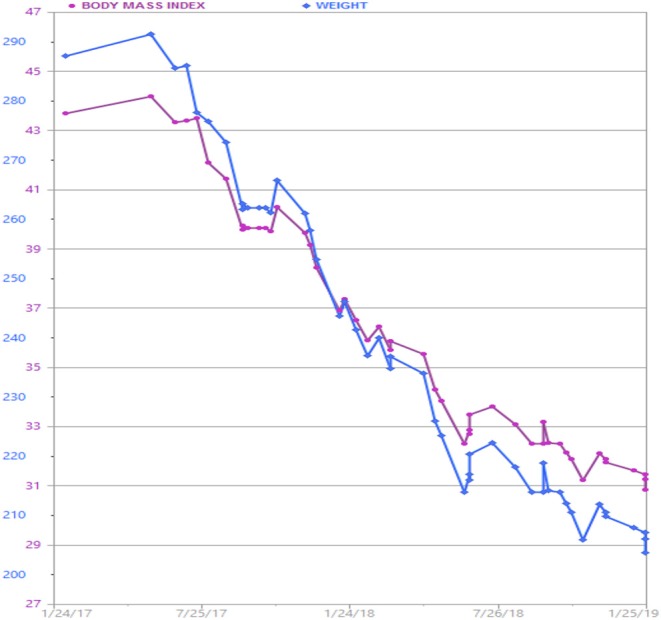
Patient's weight graph derived from the electronic health record. The patient lost a total of 90 lbs. over a 2 year time period with adjunctive anti-obesity pharmacotherapy (phentermine/topiramate) in combination with behavioral and lifestyle intervention.

The patient continued AOM long-term, having lost 90 pounds over a 2 year time period (Figure 1). Her BMI now is 28.7 kg/m^2^, weight 205 lbs. (reversed from Class 3 obesity, BMI 46 kg/m^2^, weight 295 lbs.) with improvement in quality of life and obesity-related comorbidities. Liver transaminases that were previously elevated in the context of fatty liver disease normalized along with return of regular menstrual cycles. In the process of losing weight with related attenuation in disease comorbidity and metabolic profile improvement, the patient's neurosarcoidosis continued to show remarkable recovery with stabilization of her mental health conditions and disability. Her specialists reported that this was the best she had been in many years. The patient lost −30.5% of her total body weight, which is typical weight loss achieved by metabolic and bariatric surgery means, through non-surgical intervention.

Though these results may not be usual for all patients, it is important to note that all treatment modalities (behavioral, lifestyle, pharmacological, and/or surgical whether as monotherapy or in combination) must be utilized for patients suffering with severe obesity and its devastating consequences on overall health and quality of life. Many of these patients present with complicated disease states and multiple comorbidities. Thus, important health targets include not only weight loss but treatment-enhanced double benefits leading to improvement of comorbidities.

## Data Availability Statement

All datasets for this study were directly generated from the patient's electronic health record and are available upon request.

## Informed Consent

Written informed consent to publish this case report was obtained from the patient.

## Author Contributions

GS and CA contributed and edited the contents of this manuscript.

### Conflict of Interest

GS served as a consultant for Johnson and Johnson and advisor for Rhythm Pharmaceuticals. CA reports grants from Aspire Bariatrics, Myos, the Vela Foundation, the Dr. Robert C. and Veronica Atkins Foundation, Coherence Lab, Energesis, NIH, and PCORI, grants and personal fees from Orexigen, GI Dynamics, Takeda, personal fees from Nutrisystem, Zafgen, Sanofi-Aventis, NovoNordisk, Scientific Intake, Xeno Biosciences, Rhythm Pharmaceuticals, Eisai, EnteroMedics, Bariatrix Nutrition, and other from Science-Smart LLC, outside the submitted work.

## Footnotes

^1^http://www.nationalacademies.org/hmd/~/media/Files/ActivityFiles/Nutrition/DRI-Tables/8_MacronutrientSummary.pdf?la=en (accessed April 2, 2019).
